# Multifactorial Risk Assessment of Hospital-Acquired Pressure Injuries Among High-Risk Patients: A Case–Control Study

**DOI:** 10.3390/healthcare14182895

**Published:** 2026-09-08

**Authors:** Warantron Potarin, Thidarat Somdee, Kemika Sombateyotha, Chitkamon Srichompoo, Benchaphon Aengwanich, Santisith Khiewkhern

**Affiliations:** 1 Faculty of Public Health, Mahasarakham University, Maha Sarakham 44150, Thailand; 63011490007@msu.ac.th (W.P.); thidarat@msu.ac.th (T.S.); kemika.s@msu.ac.th (K.S.); 2 Faculty of Science and Technology, Nakhon Pathom Rajabhat University, Nakhon Pathom 73000, Thailand; chitkamons@webmail.npru.ac.th; 3 Mahasarakham Hospital, Maha Sarakham 44000, Thailand; srithep.aengwanich@gmail.com

**Keywords:** hospital-acquired pressure injury, risk factors, mechanical ventilation, case–control study, pressure injury prevention

## Abstract

**Background:** Pressure injuries are a major patient safety concern, particularly among hospitalized patients at high risk. Identifying factors associated with pressure injuries is essential for effective prevention. Therefore, this study aimed to identify factors associated with pressure injuries among high-risk hospitalized patients. **Methods:** A case–control study was conducted among 80 inpatients, including 40 with hospital-acquired pressure injuries (HAPIs) and 40 without HAPIs, admitted to the Department of Medicine, Mahasarakham Hospital, between April 2023 and December 2025. Data were obtained from medical records and structured data collection forms. Descriptive statistics and bivariable analyses using chi-square or Fisher’s exact tests were performed. Variables with *p* < 0.25 were considered for multivariable logistic regression. Adjusted odds ratios (AORs) with 95% confidence intervals (CIs) were reported. **Results:** Patients with HAPIs had higher proportions of edema, incontinence, hypoalbuminemia, and nil per os (NPO) status. Bivariable analysis identified associations with Braden score ≤17, impaired level of consciousness, mechanical ventilation, inotropic/vasopressor use, physical restraint, edema, incontinence, low serum albumin, and NPO status (*p* < 0.05). In the final multivariable model, endocrine and metabolic diseases (AOR = 25.92; 95% CI: 1.06–633.10; *p* = 0.046), mechanical ventilator use (AOR = 104.79; 95% CI: 11.78–932.05; *p* < 0.001), inotropic/vasopressor use (AOR = 17.26; 95% CI: 1.15–258.41; *p* = 0.039), and diarrhea (AOR = 24.34; 95% CI: 1.88–315.36; *p* = 0.015) were statistically associated with HAPIs. Cardiovascular diseases showed a borderline association (AOR = 18.78; 95% CI: 1.00–354.36; *p* = 0.050), while physical restraint was not statistically significant (AOR = 5.40; 95% CI: 0.80–36.66; *p* = 0.084). **Conclusions:** Several clinical factors were associated with HAPIs. However, large AORs and wide CIs indicate substantial uncertainty in the magnitude of these associations. Findings should be interpreted cautiously and confirmed in larger prospective studies. Prevention should emphasize early risk identification, management of critically ill patients, and appropriate moisture and pressure injury prevention.

## 1. Introduction

Hospital-acquired pressure injuries (HAPIs), also known as pressure ulcers, remain a major global patient safety concern and a key indicator of healthcare quality. They are defined as localized damage to the skin and/or underlying tissue, typically occurring over bony prominences as a result of prolonged pressure or shear forces [1]. Despite advances in clinical care and preventive technologies, the prevalence of HAPIs in hospital settings remains substantial, with global estimates suggesting that up to 12.8% of hospitalized patients are affected, and a large proportion of these cases are hospital-acquired pressure injuries (HAPIs) [2]. Importantly, approximately 95% of HAPIs are considered preventable; however, they continue to impose significant burdens on healthcare systems, including increased morbidity, prolonged hospital stays, and higher healthcare costs [3]. Patients admitted to hospitals, particularly patients with acute or critical conditions, are at heightened risk due to multiple interacting factors such as immobility, impaired perfusion, malnutrition, and comorbidities [4].

Recent evidence has consistently identified key risk factors, including advanced age, limited mobility, incontinence, poor nutritional status, diabetes mellitus, and prolonged hospitalization [2,5]. Clinical indicators such as hypoalbuminemia, edema, and reduced functional status have also been shown to significantly increase the likelihood of HAPI development [6]. Furthermore, certain patient populations, such as those undergoing surgery or those admitted to intensive care units, are particularly vulnerable due to prolonged pressure exposure and physiological instability [7,8].

Despite extensive research, several important limitations remain. First, many studies have focused on specific subpopulations, such as surgical or elderly patients, thereby limiting the generalizability of findings to broader high-risk inpatient groups [7,9]. Second, inconsistencies in study design and outcome classification have made it difficult to distinguish hospital-acquired HAPIs from those present on admission, potentially biasing incidence estimates and associated risk factors [9]. Third, most studies have examined isolated associated factors, while fewer have comprehensively evaluated the combined effects of clinical, functional, and comorbidity-related factors within a unified analytical framework [2,6].

In addition, there is a notable lack of evidence from low- and middle-income countries, where healthcare resources, preventive practices, and patient characteristics may differ substantially from high-income settings [10]. This gap is particularly relevant in Southeast Asia, where context-specific epidemiological data remain limited. From a healthcare system perspective, HAPIs also represent a substantial economic and organizational burden, requiring intensive resource utilization for wound management, prolonged hospitalization, and increased nursing workload [3,6]. Moreover, HAPIs are widely recognized as a key quality indicator reflecting patient safety and the effectiveness of nursing care [2].

Despite these concerns, effective prevention relies heavily on accurate and timely risk assessment. However, widely used tools such as the Braden Scale have demonstrated variable predictive performance across different populations and settings [2,10]. This limitation underscores the need for context-specific evidence that integrates multiple dimensions of patient risk, including clinical, functional, and comorbidity-related factors [6,11]. Therefore, identifying independent and modifiable risk factors is essential for improving risk stratification and guiding targeted preventive interventions.

Accordingly, given the persistent burden and preventable nature of HAPIs, as well as the limitations in current evidence, particularly in resource-constrained settings, this study aims to identify and analyze factors associated with HAPIs among high-risk patients using a case–control design. By simultaneously examining demographic, clinical, and functional variables, this study seeks to determine factors independently associated with HAPI and generate context-specific evidence to enhance risk assessment, inform targeted prevention strategies, and ultimately reduce the incidence of HAPIs in hospital settings. This study aimed to compare characteristics between case and control groups in order to identify independent factors associated with HAPIs.

## 2. Methods

### 2.1. Study Design

This study employed a hospital-based case–control design to investigate factors associated with HAPIs among high-risk patients. The study was conducted at Mahasarakham Hospital, a tertiary care hospital in Thailand, and included patients admitted to selected medical wards, the stroke unit, and the medical intensive care unit (MICU). Cases were defined as patients who developed HAPIs (Stage 1 or higher) during hospitalization, whereas controls were patients who did not develop HAPIs. All pressure injuries were classified according to the 2019 international clinical practice guidelines by the European Pressure Ulcer Advisory Panel (EPUAP), National Pressure Injury Advisory Panel (NPIAP), and Pan Pacific Pressure Injury Alliance (PPIA).

### 2.2. Study Population and Sample

Population: The study population comprised patients aged 18 years or older who were admitted to the inpatient departments of Mahasarakham Hospital between April 2023 and December 2025 and were eligible for inclusion according to the predefined eligibility criteria.

Sample: The study sample consisted of eligible patients selected from the study population using a retrospective case–control design. Cases were patients who developed hospital-acquired pressure injuries (HAPIs) during hospitalization, while controls were patients who did not develop HAPIs during the same study period. All eligible cases identified during the study period were included in the study. One eligible control was selected for each case, resulting in a 1:1 case-to-control ratio [12].

#### Participant Selection

Eligible participants were identified through a retrospective review of electronic medical records from Mahasarakham Hospital between April 2023 and December 2025. Cases were adult patients aged 18 years or older who were admitted to the medical wards, stroke unit, or medical intensive care unit (MICU) and subsequently developed a hospital-acquired pressure injury (HAPI) during hospitalization. HAPI was defined as a newly developed pressure injury that was absent at admission and classified as Stage 1 or higher according to the 2019 International Guideline developed by the European Pressure Ulcer Advisory Panel (EPUAP), National Pressure Injury Advisory Panel (NPIAP), and Pan Pacific Pressure Injury Alliance (PPPIA) [13]. To ensure adequate follow-up for outcome ascertainment, case patients were required to have remained hospitalized for at least 72 h, providing sufficient observation time for identification of HAPI. Only patients with complete and accessible medical records were included. The Braden Scale score recorded at admission was collected for all participants across its full functional range (8–23) and was not used as an eligibility criterion.

All patients who met the predefined eligibility criteria for HAPI during the study period were included as cases. A total of 40 eligible cases were identified. Controls were selected from the same underlying source population as the cases and comprised adult patients aged 18 years or older who were admitted to the medical wards, stroke unit, or medical intensive care unit (MICU) during the same study period but did not develop HAPI during hospitalization. Controls were required to have remained hospitalized for at least 72 h, thereby providing a comparable observation period for pressure injury surveillance. Only patients with complete and accessible medical records were included. One eligible control was selected for each case, resulting in 40 controls and a final sample of 80 participants with a 1:1 case-to-control ratio. No individual matching by age, ward, or other characteristics was performed; instead, potential differences between cases and controls were addressed during the analytical stage using multivariable logistic regression. This approach was used to ensure that controls originated from the same source population as the cases while allowing the independent associations between candidate risk factors and HAPI to be evaluated [12].

For statistical analysis, admission Braden Scale scores were categorized using a cutoff of ≤17 to describe the distribution of participants according to their baseline clinical vulnerability. Scores ≤ 17 were classified as “at risk,” whereas scores > 17 were classified as “low risk.” This categorization was used solely for descriptive and analytical purposes and did not constitute an inclusion or exclusion criterion. Potential confounding related to differences in baseline disease severity and care intensity across clinical settings was addressed using multivariable logistic regression.

### 2.3. Outcome Measures

The primary outcome was the occurrence of HAPIs, defined as the development of a new HAPI during hospitalization that was not present at the time of admission. Pressure injuries were identified and staged (Stage 1 or higher) based on clinical documentation and classified according to the 2019 international clinical practice guidelines by EPUAP, NPIAP, and PPPIA. The outcome was treated as a binary variable (presence vs. absence of HAPIs), defined as a binary variable (yes/no).

Independent variables included demographic and clinical characteristics: age, sex, underlying diseases (e.g., diabetes mellitus, hypertension), length of hospital stay, Braden Scale score, mobility status, nutritional status (e.g., serum albumin level), presence of edema, incontinence, use of medical devices, and level of consciousness.

Potential confounding variables were identified based on prior literature and clinical relevance, including age, comorbidities, nutritional status, and severity-related indicators. These variables were considered in the multivariable analysis to control for their potential confounding effects on the association between exposure variables and HAPIs.

### 2.4. Instrument Validity and Reliability

A structured data abstraction form comprising 110 items was developed to systematically extract demographic, clinical, and treatment-related information from patients’ medical records. The instrument was designed based on an extensive review of the literature, clinical practice guidelines, and variables identified as potential risk factors for pressure injuries in critically ill patients.

Content validity was established through independent review by a panel of three experts with expertise in critical care nursing, wound care, and epidemiology. Each item was evaluated using the Item-Objective Congruence (IOC) method, and all items achieved IOC values greater than 0.50, indicating acceptable content validity. The instrument was subsequently revised according to the experts’ recommendations to improve clarity and relevance before pilot testing.

Prior to the main study, the revised data abstraction form was pilot-tested using 30 medical records from patients with characteristics comparable to those of the study population. The pilot study was conducted to evaluate the feasibility, clarity, and consistency of the data collection procedures. Internal consistency reliability was assessed using Cronbach’s alpha coefficient, yielding a value of 0.89, indicating good reliability. Standardized data collection procedures were implemented throughout the study to ensure consistency in data abstraction.

To ensure consistency in data abstraction, all data collectors received standardized training before data collection. A detailed data abstraction manual was used throughout the study, and any discrepancies were resolved through discussion and consensus.

### 2.5. Data Collection Procedures

Data were collected through a retrospective review of electronic and paper-based medical records of eligible patients admitted to Mahasarakham Hospital between April 2023 and December 2025. Clinical information was extracted using a standardized data abstraction form developed specifically for this study. The form was designed to ensure consistent collection of demographic characteristics, clinical conditions, treatment-related variables, laboratory findings, and pressure injury outcomes according to predefined operational definitions.

Data abstraction was performed by trained research personnel with experience in medical record review. Prior to data collection, all reviewers received standardized training on study procedures, variable definitions, and the use of the data abstraction form to ensure consistency in data collection. Patient information was systematically extracted from electronic medical records, nursing documentation, physician progress notes, laboratory reports, medication administration records, and other relevant clinical documentation. To establish a proper temporal relationship and ensure that dynamic clinical exposures were evaluated as associated factors rather than consequences of greater disease severity, a strict chronological data extraction protocol utilizing explicit temporal anchors was enforced. For the case group, an “Index Date” was defined as the exact date and time of the initial confirmed onset of the hospital-acquired pressure injury (HAPI). All independent clinical variables—including mechanical ventilation, physical restraint, NPO status, diarrhea, urinary and fecal incontinence, edema, serum albumin, vasopressor/inotropic therapy, and Braden Scale scores—were assessed according to their documented status strictly prior to this Index Date. Any clinical exposure initiated after or simultaneously with the detection of the first pressure injury was excluded from the analysis for that participant. For the control group, a methodologically equivalent observation window was established by applying a “Reference Time” cutoff based on the median duration from admission to HAPI onset observed in the case cohort, which was determined to be 4 days (96 h). Consequently, clinical variables for control participants were systematically audited and coded according to their documented status within the initial 96 h following hospital admission [14]. Any clinical event or intervention initiated after the Index Date for cases or after the 96 h Reference Time for controls was excluded.

The occurrence, onset, anatomical location, and stage of hospital-acquired pressure injuries were verified using nursing and physician documentation and classified according to the 2019 International Clinical Practice Guideline developed by the European Pressure Ulcer Advisory Panel (EPUAP), National Pressure Injury Advisory Panel (NPIAP), and Pan Pacific Pressure Injury Alliance (PPPIA) [1,13]. Pressure injuries were considered hospital-acquired only when documentation confirmed that no pressure injury was present at admission and that the lesion developed during hospitalization. The exact timestamps of the initial HAPI discovery were carefully cross-referenced against the timeline of clinical interventions to guarantee accurate temporal ordering.

To enhance data quality and minimize information bias, all data were abstracted according to predefined operational definitions. Records containing incomplete or potentially inconsistent information were independently reviewed by a second investigator, and discrepancies were resolved through discussion and consensus. In addition, periodic quality checks were performed throughout the data collection process to verify the completeness, consistency, and accuracy of the extracted data before statistical analysis.

### 2.6. Statistical Analysis

Data were analyzed using IBM SPSS Statistics version 24.0 (IBM Corp., Armonk, NY, USA). Descriptive statistics were used to summarize participant characteristics. Categorical variables were presented as frequencies and percentages, whereas continuous variables were summarized as means and standard deviations (SDs) or medians with interquartile ranges (IQRs), as appropriate, according to data distribution.

Differences in baseline characteristics between patients with and without hospital-acquired pressure injuries (HAPIs) were initially assessed using the chi-square test or Fisher’s exact test for categorical variables and the independent-samples t-test or Mann–Whitney U test for continuous variables, as appropriate. Univariable logistic regression analyses were subsequently performed to estimate crude odds ratios (ORs) and 95% confidence intervals (CIs) for potential factors associated with HAPIs. Variables with a *p*-value < 0.25 in the univariable analysis, together with variables considered clinically important based on previous evidence, were considered for multivariable analysis. Initially, 13 clinically relevant candidate variables were considered, including patient status, Braden score at admission, length of stay, endocrine/metabolic disease, cardiovascular disease, physical restraint, mechanical ventilation, inotropic drug use, temperature at admission, incontinence, edema, diarrhea, and level of consciousness.

Multivariable logistic regression analysis was performed using a backward stepwise procedure based on the Wald statistic to identify factors independently associated with HAPIs while accounting for potential confounding. Variables were retained according to the predefined statistical criteria of *p* < 0.05 for entry and *p* > 0.10 for removal. Adjusted odds ratios (AORs) with corresponding 95% confidence intervals (CIs) were reported to quantify the strength and precision of the associations. Given the relatively small number of HAPI cases (*n* = 40) and the presence of sparse data cells for some exposure categories, the multivariable estimates were interpreted cautiously, particularly where large effect estimates and wide confidence intervals were observed. Multicollinearity among independent variables was assessed using variance inflation factors (VIFs), with VIF values < 5 indicating no evidence of problematic multicollinearity; the maximum VIF observed in the final model was 4.1. Model calibration was assessed using the Hosmer–Lemeshow goodness-of-fit test, which indicated no evidence of poor model fit (Chi-square = 0.754, *p* = 0.993). Model explanatory performance was additionally assessed using the Nagelkerke R Square, which was 0.721, and overall classification accuracy was 87.50%. These model-performance measures were interpreted cautiously because of the limited number of HAPI cases, sparse exposure categories, and potential model instability or overfitting. The analysis was intended to identify factors potentially associated with HAPIs rather than to develop or validate a clinical prediction model. All analyses were based on complete-case data, and a two-sided *p*-value < 0.05 was considered statistically significant.

### 2.7. Ethical Considerations and Informed Consent

The study protocol was reviewed and approved by the Human Research Ethics Committee of Mahasarakham University (Approval No. 273/2566). As this study involved a retrospective review of existing medical records and posed minimal risk to participants, the requirement for informed consent was waived by the Ethics Committee. All patient data were anonymized prior to analysis to ensure confidentiality and privacy. The study was conducted in accordance with the ethical principles of the Declaration of Helsinki and all applicable institutional guidelines and regulations.

## 3. Results

### 3.1. Demographic of the Participants

#### 3.1.1. Demographic Characteristics

The demographic profile of the participants revealed that the majority were male (58.80%) while females accounted for 41.20%. When comparing the two groups, the proportion of males was higher in the HAPI group (62.50%) than in the non-HAPI group (55.00%). Regarding education, 96.20% of the total sample had attained at least a primary education level, while 3.80% had no formal education. Employment status was nearly equally distributed, with 51.20% employed and 48.80% unemployed. In terms of marital status, the majority were married or had a partner; however, this was more prevalent in the HAPI group (82.50%) compared to the non-HAPI group (52.50%).

Using the income threshold of 3078 THB per month [15] most participants (67.50%) earned above this criterion. The mean monthly income for the HAPI group was 5580 ± 4967.55 THB while the non-HAPI group reported a higher mean income of 6930 ± 5479.34 THB. Furthermore, 85.00% of the total sample reported underlying medical conditions, specifically 82.50% in the HAPI group and 87.50% in the non-HAPI group.

#### 3.1.2. Clinical Characteristics and Braden Scale Scores

Assessment of the Braden Scale at admission showed a mean score of 13.60 ± 4.18 for the HAPI group and 15.55 ± 3.74 for the non-HAPI group. The overall mean score for all participants was 14.01 ± 2.63 (range: 8–23). On the date of data collection, the HAPI group had a significantly lower mean Braden Scale score of 12.30 ± 2.43 (range: 8–16) compared with the non-HAPI group, which had a mean score of 15.73 ± 1.88 (range: 10–18).

When participants were categorized using a Braden Scale cutoff of ≤17, 80.00% of the HAPI group and 67.50% of the non-HAPI group had scores classified as “at risk” at admission. By the date of data collection, the proportion of participants classified as “at risk” was 100.00% in the HAPI group and 82.50% in the non-HAPI group.

#### 3.1.3. Physical Status and Diagnosis

Regarding the level of consciousness, 80.00% of the HAPI group were fully conscious, while 8.00% were unconscious. In the non-HAPI group, 97.50% were fully conscious and 2.50% were unconscious. The Body Mass Index (BMI) analysis showed that both groups had an identical proportion of individuals with a BMI below 23 kg/m^2^ (52.50%). The overall mean BMI was 23.45 ± 5.68 kg/m^2^ (range: 9.91–40.00 kg/m^2^).

Primary diagnoses during hospitalization were similar across both groups. The most common conditions were infectious diseases (HAPI group: 60.00%; non-HAPI group: 56.50%), followed by respiratory diseases (HAPI group: 45.00%; non-HAPI group: 47.50%) and renal/urinary tract diseases (HAPI group: 25.00%; non-HAPI group: 32.50%). Detailed data are presented in Table 1.

### 3.2. Mobility and Activity Factors

Regarding the length of hospital stay, the majority of the total study population (66.20%) remained hospitalized for 8 days or longer. Within the HAPI group, 73.30% had a stay of at least 8 days, with a mean duration of 17.25 ± 12.98 days. In comparison, 60.00% of the non-HAPI group had a similarly long stay although the mean duration of their stay was slightly shorter at 14.40 ± 28.52 days.

In terms of medical interventions and physical restrictions, patients in the HAPI group showed higher rates of receiving intravenous (IV) medications and being restrained. Specifically, 7.50% of the HAPI group received IV sedation, and 22.50% received IV analgesics, whereas none of the patients in the non-HAPI group (0.00%) received either type of medication. Furthermore, 60.00% of patients who developed pressure injuries were subject to physical restraint, which was significantly higher than the 17.50% observed in the non-HAPI group.

The assessment of functional status through Activities of Daily Living (ADL) scores revealed a marked difference between the two groups. The HAPI group had a significantly lower mean ADL score of 3.05 ± 2.76 compared to 6.30 ± 5.46 in the non-HAPI group. When categorized by dependency levels, all participants in the HAPI group (100.00%) were classified as “Dependent” (scoring less than 12 points). While dependency was also high in the non-HAPI group (85.00%), a small portion of that group (15.00%) remained “Independent”, as presented in Table 2.

### 3.3. Oxygen Perfusion Factors

This study compared factors related to oxygen perfusion between the HAPI group and the non-HAPI group. One of the most significant differences was found in the use of life-sustaining equipment; 77.50% of patients in the HAPI group required a mechanical ventilator, whereas only 7.50% of those in the non-HAPI group used one. Similarly, the administration of inotropic or vasopressor drugs was more frequent in the HAPI group (27.50%) compared to the non-HAPI group (7.50%). Despite these clinical differences, both groups maintained a Mean Arterial Pressure of 65 mmHg or higher at 100%, with mean values of 91.55 ± 15.06 mmHg for the HAPI group and 94.08 ± 16.33 mmHg for the non-HAPI group.

Regarding comorbidities and clinical indicators, the prevalence of diabetes mellitus was relatively similar between the HAPI (30.00%) and non-HAPI (32.50%) groups, while vascular diseases affected exactly 50.00% of participants in both groups. Capillary blood glucose levels were also consistent across both categories, with 77.50% of patients in each group maintaining levels at or below 180 mg%. However, smoking history was more prevalent among those who developed HAPIs, with 62.50% being smokers compared to 42.50% in the non-HAPI group.

Finally, the study assessed hemoglobin levels using a threshold of 11 g/dL. It was found that 57.50% of the HAPI group and 65.00% of the non-HAPI group had hemoglobin levels ≤ 11 g/dL. The mean hemoglobin level for the HAPI group was 10.36 ± 2.43 g/dL while the non-HAPI group showed a slightly lower mean of 10.18 ± 2.80 g/dL. Overall, these findings highlight that patient requiring higher levels of clinical support, such as using ventilation and vasopressors, were more frequently found in the group that developed HAPIs, as presented in Table 3.

### 3.4. Skin Integrity and Nutritional Factors

A comparison of demographic and clinical characteristics between patients with and without HAPIs was undertaken. The majority of participants in both groups were aged over 60 years, accounting for 65.00% in the HAPI group and 67.50% in the non-HAPI group. The mean age was slightly higher in the HAPI group (66.75 ± 12.31 years) compared to the non-HAPI group (62.98 ± 17.11 years).

Regarding body temperature, a higher proportion of patients in the HAPI group had a temperature of ≥37.5 °C (55.00%) compared to those without HAPIs (37.50%). The mean body temperature was also higher in the HAPI group (37.67 ± 1.16 °C vs. 37.28 ± 1.06 °C).

Edema was more prevalent among patients with pressure injuries (62.50%) than those without (32.5%). Similarly, urinary incontinence was highly prevalent in both groups but was more common in the HAPI group (95.00% vs. 75.00%). Fecal incontinence also showed a notable difference, with 75.00% in the HAPI group compared to 32.50% in the non-HAPI group.

In terms of nutritional status, most patients in both groups had serum albumin levels of ≤3.5 g/dL; however, this was more pronounced in the HAPI group (97.5%) compared to the non-HAPI group (82.50%). The mean serum albumin level was lower in the HAPI group (2.45 ± 0.44 g/dL) than in the non-HAPI group (2.92 ± 0.58 g/dL).

Finally, oral intake status differed markedly between groups. A substantially higher proportion of patients in the HAPI group were on nil per os (NPO) status (65.00%) compared to only 7.50% in the non-HAPI group, whereas normal oral intake was more common among patients without HAPIs (92.50%), as presented in Table 4.

### 3.5. Factors Associated with Pressure Injuries (Bivariate Analysis)

The bivariate analysis identified several factors significantly associated with the occurrence of HAPIs. Marital status demonstrated a statistically significant association, with a higher proportion of patients who were married or partnered in the HAPI group compared to the non-HAPI group (82.50% vs. 52.50%; crude OR = 4.26, *p* = 0.004).

Patients classified as being at risk based on the Braden Scale (score ≤ 17) were significantly more likely to develop HAPIs than those at low risk (97.50% vs. 82.50%; crude OR = 8.27, *p* = 0.011). Similarly, patients with impaired consciousness had significantly higher odds of acquiring a HAPI compared to those who were conscious (20.00% vs. 2.50%; crude OR = 9.75, *p* = 0.031).

Clinical intervention-related factors showed strong associations. The use of mechanical ventilation was markedly higher among patients with HAPIs (77.50% vs. 7.50%), representing the strongest association observed in the bivariate analysis (crude OR = 42.48, *p* < 0.001). In addition, patients who were physically restrained had significantly higher odds of developing HAPIs compared to those who were not restrained (60.00% vs. 17.50%; crude OR = 7.07, *p* < 0.001). The use of inotropic or vasopressor drugs was also significantly associated with HAPI occurrence (27.50% vs. 7.50%; crude OR = 4.68, *p* = 0.037).

Patient condition-related factors were also significant. The presence of edema was associated with increased odds of HAPI (62.50% vs. 32.50%; crude OR = 3.46, *p* = 0.013). Urinary and fecal incontinence were more prevalent among patients with HAPIs, with fecal incontinence showing a particularly strong association (75.00% vs. 32.50%; crude OR = 6.23, *p* < 0.001).

Nutritional status was another important determinant. Patients with low serum albumin levels (≤3.5 g/dL) had significantly higher odds of developing HAPIs compared to those with normal levels (97.50% vs. 82.50%; crude OR = 8.27, *p* = 0.038). Furthermore, patients who were on NPO status were substantially more likely to develop HAPIs than those with normal oral intake (65.00% vs. 7.50%; crude OR = 22.90, *p* < 0.001).

Overall, the findings indicate that factors related to impaired mobility, severity of illness, moisture exposure, and poor nutritional status were significantly associated with the development of HAPIs in the bivariate analysis, as presented in Table 5.

### 3.6. Factors Associated with Pressure Injuries (Multivariate Analysis)

Six factors were retained in the final multivariable logistic regression model: endocrine and metabolic diseases, cardiovascular diseases, physical restraint, mechanical ventilator use, inotropic/vasopressor use, and diarrhea. After adjustment for the other factors in the final model, endocrine and metabolic diseases were significantly associated with HAPIs (AOR = 25.92, 95% CI: 1.06–633.10, *p* = 0.046). Mechanical ventilator use showed a strong association with HAPIs (AOR = 104.79, 95% CI: 11.78–932.05, *p* < 0.001). Inotropic/vasopressor use was also significantly associated with HAPIs (AOR = 17.26, 95% CI: 1.15–258.41, *p* = 0.039), as was diarrhea (AOR = 24.34, 95% CI: 1.88–315.36, *p* = 0.015).

Cardiovascular diseases showed a borderline association with HAPIs (AOR = 18.78, 95% CI: 1.00–354.36, *p* = 0.050), whereas physical restraint was not statistically significant after adjustment (AOR = 5.40, 95% CI: 0.80–36.66, *p* = 0.084). The final model demonstrated good agreement between the observed and expected outcomes based on the Hosmer–Lemeshow goodness-of-fit test (χ^2^ = 0.754, *p* = 0.993). The model had a Nagelkerke R^2^ of 0.721 and correctly classified 87.5% of observations, as shown in Table 6.

## 4. Discussion

Hospital-acquired pressure injury (HAPI) remains a major preventable adverse event among hospitalized patients and is widely recognized as an important nursing-sensitive indicator reflecting the quality of patient surveillance, preventive care, and multidisciplinary clinical management [3,7,16]. The present study investigated factors associated with HAPI development among hospitalized patients with elevated vulnerability and found that HAPI occurrence was associated with a complex combination of patient vulnerability, underlying clinical conditions, and treatment-related factors. Although several demographic and clinical characteristics were associated with HAPI development in the initial analysis, six factors remained in the final multivariable logistic regression model: endocrine and metabolic diseases, cardiovascular diseases, physical restraint, mechanical ventilator use, inotropic/vasopressor use, and diarrhea. Among these factors, endocrine and metabolic diseases, mechanical ventilator use, inotropic/vasopressor use, and diarrhea were statistically associated with HAPIs after adjustment, whereas cardiovascular diseases showed a borderline association, and physical restraint was not statistically significant. These findings suggest that HAPI development is unlikely to be attributable to a single factor but may reflect the interplay of impaired mobility, reduced tissue tolerance, altered perfusion, moisture exposure, and systemic physiological disturbances [16,17,18]. The final multivariable model showed acceptable calibration based on the Hosmer–Lemeshow test; however, these findings should be interpreted cautiously given the small number of HAPI cases, sparse exposure frequencies, and potential model instability. Further studies with larger samples and more HAPI events are warranted to confirm the observed associations.

The demographic characteristics of this study population provide important context for interpreting the findings. Most participants were older adults, with a mean age of approximately 65 years, reflecting the high prevalence of age-related vulnerability among hospitalized patients at risk for HAPI. Aging is traditionally considered an important risk factor because it is associated with structural and functional changes in the skin, including reduced dermal thickness, decreased collagen synthesis, diminished elasticity, impaired microvascular function, and delayed tissue repair capacity [16,19,20]. However, age was not independently associated with HAPI development after controlling for clinical and treatment-related factors in this study. This finding suggests that chronological age alone may be insufficient to explain HAPI occurrence in populations where advanced age is common. Instead, age may function as a background vulnerability factor that increases susceptibility when combined with acute illness, immobility, impaired circulation, or intensive care interventions. Recent evidence similarly indicates that functional dependency, frailty, illness severity, and treatment intensity may have stronger predictive value than age alone in hospitalized and critically ill populations [21,22].

The absence of an independent association between age and HAPI development has important clinical implications. Older adults should not be considered particularly vulnerable solely because of age; rather, clinical assessment should consider physiological status, mobility limitations, tissue perfusion, nutritional condition, and exposure to external pressure. This approach is consistent with current recommendations emphasizing individualized assessment of patient vulnerability rather than relying on demographic characteristics alone [13,18]. In clinical practice, older patients with preserved mobility and stable physiological conditions may have fewer characteristics associated with HAPI than younger patients experiencing severe critical illness requiring invasive interventions. Therefore, prevention strategies should consider the overall profile of modifiable and non-modifiable factors associated with HAPI.

Among the factors independently associated with HAPI identified in this study, mechanical ventilation demonstrated the strongest adjusted association (AOR = 104.79, 95% CI: 11.78–932.05, *p* < 0.001). However, the remarkably large numerical magnitude of this AOR and its wide confidence interval should be interpreted with caution, as they indicate substantial statistical imprecision and potential model instability rather than a precise estimate of the strength of association. This wide interval may occur in multivariable analyses with a limited sample size when an exposure is highly concentrated in one group, as observed in this study, where mechanical ventilation was present in 77.50% of cases compared with 7.50% of controls. Therefore, the finding should not be interpreted solely on the basis of the magnitude of the AOR but should be considered in the context of the overall clinical pattern and the limitations of the model. The observed association is also consistent with previous evidence indicating that mechanically ventilated patients frequently have characteristics associated with HAPI, including immobility, sedation, impaired sensory perception, and physiological instability. Mechanical ventilation itself should not be interpreted as directly causing tissue injury; rather, it may serve as a clinical marker of severe illness and a combination of conditions associated with greater vulnerability to pressure-related tissue damage [1,18].

Patients receiving mechanical ventilation commonly receive sedation, analgesia, or neuromuscular blockade, which may be associated with reduced spontaneous movement and limited ability to independently reposition the body. Prolonged pressure over bony prominences may compromise capillary blood flow and is associated with tissue ischemia, cellular hypoxia, inflammation, and progressive tissue damage. In addition, critically ill patients receiving mechanical ventilation frequently experience impaired oxygen delivery, systemic inflammatory responses, hemodynamic instability, and altered microcirculation, which may be associated with reduced tissue tolerance to mechanical loading. Therefore, the strong association observed in this study may reflect the combined presence of immobility and critical illness-related physiological impairment rather than mechanical ventilation alone.

Despite the statistical uncertainty surrounding the exact numerical strength of this association, the findings highlight the importance of considering mechanically ventilated patients as a clinically vulnerable population in HAPI prevention. This interpretation is consistent with evidence from systematic reviews examining targeted preventive approaches in critical care settings. For example, systematic evidence suggests that the use of constant low-pressure or alternating-pressure redistribution surfaces may help limit tissue deformation and pressure-related tissue compromise among immobile patients compared with standard hospital mattresses [1,4,23]. Furthermore, systematic evidence suggests that multilayer silicone-bordered prophylactic foam dressings may help preserve skin barrier integrity and may be associated with lower HAPI occurrence among vulnerable hospitalized patients [3,13,18]. In mechanically ventilated patients, who may be particularly vulnerable to pressure and tissue deformation associated with medical device securement, meta-analytic evidence suggests that standardized endotracheal tube stabilization devices and nasal alar barrier dressings may be associated with fewer device-related pressure injuries [1,18,24].

To effectively translate these individual interventions into actionable clinical practice, recent comprehensive systematic reviews support the implementation of structured, multifaceted pressure injury prevention care bundles incorporating standardized clinical assessment, frequent skin assessments, specialized positioning devices, and nutritional optimization [2,6,25]. These systematic evaluations have reported reductions in HAPI incidence ranging from 4.3% to 36.2% following implementation of multifaceted prevention approaches across diverse healthcare settings, with some studies also reporting shorter hospital stays and lower wound severity [2,8,24]. The effectiveness of these approaches may be enhanced through multidisciplinary coordination and regular nursing staff training [3,24,25]. Therefore, rather than relying on uniform, non-specific protocols, integrating mechanical ventilation status into structured, multidisciplinary nursing prevention bundles and using appropriate pressure redistribution and prophylactic technologies may support more individualized HAPI prevention among clinically vulnerable patients in acute care settings [2,18,26].

Physical restraint was associated with HAPI occurrence in the unadjusted analysis; however, the association was attenuated after adjustment and did not reach statistical significance in the final model (AOR = 5.40, 95% CI: 0.80–36.66, *p* = 0.084). This finding should not be interpreted as evidence that physical restraint is clinically irrelevant. Rather, the observed association may be partly explained by the underlying clinical conditions that lead to restraint use, including impaired consciousness, severe illness, mechanical ventilation, or limited mobility [25,27,28]. Physical restraint may also restrict spontaneous repositioning and pressure redistribution, potentially increasing exposure to prolonged mechanical loading. However, because the confidence interval was wide and included the null value, the present study does not provide sufficient statistical evidence to conclude that physical restraint is independently associated with HAPI development.

Beyond the mechanical effects, restraint use may be associated with delayed recognition of early signs of pressure-related discomfort or skin changes. Patients who are sedated, cognitively impaired, or unable to communicate symptoms may remain exposed to prolonged pressure when position adjustments are limited or delayed. Recent nursing literature emphasizes that restraint use should be minimized whenever possible through alternative safety strategies, including close observation, individualized care planning, family involvement, delirium prevention, and early mobilization approaches [25,27,28]. Therefore, minimizing unnecessary restraint use remains an important ethical and patient-centered component of comprehensive HAPI prevention, even though the present study did not demonstrate a statistically significant independent association.

The identification of diarrhea as a factor independently associated with HAPI development (AOR = 24.34, 95% CI: 1.88–315.36, *p* = 0.015) highlights the potential importance of moisture-associated skin damage in the development of HAPI. Although mechanical pressure and shear remain important forces associated with tissue deformation, excessive moisture may compromise the skin’s resistance to these forces by disrupting epidermal barrier integrity and increasing susceptibility to friction and injury [29,30]. Diarrhea may be particularly concerning because repeated exposure to liquid stool involves contact with digestive enzymes, microorganisms, and other irritant substances that may contribute to skin maceration and inflammation.

This finding is clinically important because diarrhea represents a potentially modifiable factor associated with HAPI through appropriate nursing management. Patients with frequent diarrhea may benefit from prompt cleansing, appropriate barrier protection, moisture control strategies, regular skin inspection, and individualized prevention plans. These measures may be particularly relevant among critically ill patients, who may also have compromised skin integrity associated with immobility, inflammation, and reduced physiological reserve [18,26,29]. The present findings support the integration of moisture management into comprehensive HAPI prevention bundles rather than treating it as a secondary nursing concern. Nevertheless, the large AOR and wide confidence interval observed in this study indicate substantial uncertainty regarding the magnitude of this association.

Endocrine and metabolic diseases were independently associated with HAPI development (AOR = 25.92, 95% CI: 1.06–633.10, *p* = 0.046). Although this category includes multiple disorders, diabetes mellitus is likely an important contributor because of its well-established effects on vascular function, tissue repair, and wound healing. Chronic hyperglycemia is associated with endothelial dysfunction, impaired microcirculation, peripheral neuropathy, reduced immune response, and delayed collagen formation, which may decrease tissue resistance to prolonged pressure exposure [20,31,32].

However, the very wide confidence interval observed for this variable indicates considerable uncertainty regarding the magnitude of the association. This may reflect the relatively small number of patients with endocrine or metabolic diseases or heterogeneity among the underlying conditions. Therefore, this finding should be interpreted cautiously. Nevertheless, the biological plausibility of the observed association and its consistency with previous evidence support the importance of careful skin monitoring and preventive care among patients with metabolic disorders [31,32]. Future studies with larger sample sizes should further examine specific metabolic conditions and their independent associations with HAPI development.

Cardiovascular diseases showed a borderline association with HAPI development after multivariable adjustment (AOR = 18.78, 95% CI: 1.00–354.36, *p* = 0.050). The direction and magnitude of the estimate suggest a potentially important clinical association; however, the confidence interval was extremely wide and the *p*-value was exactly at the conventional threshold for statistical significance. Therefore, this finding should be interpreted as borderline rather than as definitive evidence of an independent association. Cardiovascular disease may contribute to HAPI vulnerability through impaired circulation, reduced tissue oxygenation, hemodynamic instability, and reduced physiological reserve [20,21]. Further studies with larger samples are needed to determine whether this association is reproducible.

Inotropic/vasopressor use was also independently associated with HAPI development in the final model (AOR = 17.26, 95% CI: 1.15–258.41, *p* = 0.039). This association is biologically plausible because vasopressor therapy may increase peripheral vasoconstriction and potentially reduce tissue perfusion, particularly in patients with severe hemodynamic instability. However, the wide confidence interval indicates substantial uncertainty regarding the magnitude of the association. In addition, vasopressor use frequently occurs alongside other markers of critical illness, including mechanical ventilation, septic shock, and multiple organ dysfunction. Thus, the observed association may reflect both the physiological effects of vasoactive therapy and the underlying severity of illness. Previous studies have reported variable associations between vasopressor exposure and HAPI development, suggesting that its role should be considered within the broader context of hemodynamic status and overall illness severity [1,21].

Although several variables were significantly associated with HAPI development in the bivariate analysis, including low Braden Scale scores, edema, urinary and fecal incontinence, hypoalbuminemia, impaired consciousness, vasopressor administration, and NPO status, these factors were not all retained as statistically significant factors in the final multivariable model. This finding should not be interpreted as indicating that these factors are clinically irrelevant. Rather, it may reflect the complex interrelationships among factors associated with HAPI in hospitalized patients, particularly those with severe illness. Many of these variables may represent overlapping pathways or clinical conditions related to illness severity that are associated with increased tissue vulnerability [17,21,22,23].

The Braden Scale remains one of the most widely used instruments for assessing HAPI risk and incorporates six domains related to sensory perception, moisture, activity, mobility, nutrition, and friction/shear [16,17]. In this study, patients who developed HAPI had significantly lower Braden scores than those without HAPI in the bivariate analysis, highlighting the potential clinical relevance of comprehensive risk assessment. However, the Braden score was not retained in the final multivariable model. This finding may reflect substantial overlap between the domains assessed by the Braden Scale and the clinical factors associated with HAPI in this study. For example, mechanical ventilation may be associated with impaired mobility and altered sensory responses, diarrhea is associated with increased moisture exposure and skin vulnerability, and endocrine or metabolic disorders may be associated with nutritional status and tissue-healing capacity. Thus, the association between Braden Scale scores and HAPI development observed in the bivariate analysis may have been attenuated after adjustment for other clinical factors [18,19,24].

Nevertheless, these findings do not diminish the importance of Braden Scale assessment in clinical practice. Risk assessment tools are designed primarily for the early identification of patients who may be vulnerable to HAPI and should be interpreted alongside clinical judgment rather than used as standalone assessment tools. Previous studies have suggested that combining standardized risk assessment instruments with objective clinical variables, intensive care interventions, and factors identified from electronic health records may improve the identification of patients at risk for hospital-acquired HAPI [21,22,27]. Therefore, routine Braden Scale assessment remains an important component of nursing practice, particularly when combined with ongoing evaluation of changes in patients’ clinical conditions.

Nutritional status represents another important component of HAPI prevention. In the present study, patients with HAPI had lower serum albumin levels and were more likely to have NPO status during hospitalization. These findings are consistent with previous evidence indicating that nutritional compromise is associated with reduced tissue tolerance, impaired collagen synthesis, delayed wound healing, and increased susceptibility to pressure-related injury [20,33]. However, hypoalbuminemia and NPO status were not retained as statistically significant factors in the final multivariable model. This may be because serum albumin is influenced by multiple factors beyond nutritional intake, including systemic inflammation, infection, fluid imbalance, and acute disease severity. Similarly, NPO status often reflects underlying clinical complexity, such as respiratory failure, surgical conditions, or gastrointestinal dysfunction, rather than representing a factor independently associated with HAPI development [21,22].

Despite the lack of independent statistical significance, nutritional assessment and optimization remain important components of HAPI prevention. Current recommendations emphasize that nutritional interventions should be incorporated into comprehensive prevention programs, particularly among patients with prolonged hospitalization, critical illness, or evidence of nutritional risk [13,18]. Early nutritional screening, appropriate feeding strategies, and collaboration with dietitians may support tissue resilience and recovery among patients at elevated risk of HAPI.

Edema and incontinence also demonstrated significant associations with HAPI occurrence in the unadjusted analysis but were not retained in the final multivariable model. Edema is associated with increased interstitial pressure, reduced capillary perfusion, impaired oxygen diffusion, and decreased tolerance to external loading [19,29]. Similarly, urinary and fecal incontinence are associated with increased moisture exposure, disruption of the skin barrier, and friction-related skin injury. Fecal incontinence may be of particular concern because digestive enzymes and microorganisms present in stool may be associated with inflammation and epidermal breakdown [29,30]. However, these conditions frequently occur together with immobility, mechanical ventilation, prolonged hospitalization, and severe illness. Therefore, the observed associations of edema and incontinence with HAPI in the unadjusted analysis may reflect their interrelationships with other clinical factors rather than indicating isolated independent associations [23,24].

Overall, these findings demonstrate the importance of distinguishing statistical independence from clinical importance. Variables that were significant in the bivariate analysis but were not retained as statistically significant in the final model may still provide valuable information regarding patient vulnerability and should continue to inform nursing surveillance. In complex clinical populations, HAPI prevention requires consideration of multiple interacting clinical factors rather than reliance on a single associated factor. A patient-centered approach should integrate risk assessment tools, clinical observation, and evaluation of treatment-related exposures to identify individuals who may require intensified prevention strategies [13,18,19].

From a nursing practice perspective, the findings highlight several important implications. First, HAPI prevention should begin with early identification of patients exposed to clinical conditions and interventions associated with increased vulnerability, particularly mechanical ventilation and hemodynamic support. These patients may benefit from proactive prevention strategies rather than waiting for early skin changes to appear. Regular head-to-toe skin assessment, evaluation of pressure points, monitoring of medical device-related pressure, and individualized repositioning schedules should be incorporated into routine care for critically ill patients [13,18].

To optimize the translation of these findings into clinical practice, bedside clinicians should carefully distinguish between modifiable factors associated with HAPI and non-modifiable markers of underlying disease severity. In this study, mechanical ventilation (AOR = 104.79), endocrine and metabolic diseases (AOR = 25.92), inotropic/vasopressor use (AOR = 17.26), and diarrhea (AOR = 24.34) were statistically associated with HAPI in the final model. However, the large effect estimates and wide confidence intervals indicate considerable uncertainty regarding their precise magnitude. These factors may therefore be viewed as clinical indicators of greater physiological vulnerability rather than as definitive causal determinants. Their presence at the bedside may nevertheless warrant increased clinical vigilance. Rather than relying solely on periodic scoring systems, patients receiving mechanical ventilation or hemodynamic support may benefit from closer nursing surveillance, including assessment of pressure redistribution needs and monitoring for pressure-related injury associated with immobility or medical devices.

Conversely, diarrhea represents a potentially modifiable factor associated with HAPI and may provide opportunities for individualized nursing care. Patients with diarrhea may benefit from early measures to prevent moisture-associated skin damage (MASD), including appropriate skin protectants, structured cleansing regimens, and regular assessment of the skin and microclimate. Although physical restraint was not statistically significant after adjustment, minimizing unnecessary restraint use remains appropriate because of its potential effects on mobility and patient-centered care. Integrating these clinical factors into routine bedside assessment alongside standard measures such as the Braden Scale may support more individualized and dynamic HAPI prevention strategies based on each patient’s evolving clinical condition.

Second, preventive interventions should be individualized according to the patient’s dominant vulnerability profile. Patients receiving mechanical ventilation may benefit from enhanced pressure redistribution, appropriate support surfaces, and close monitoring of immobility-related risks. Patients experiencing diarrhea may benefit from proactive moisture management, barrier protection, and frequent skin reassessment. Those with endocrine or metabolic diseases may warrant closer monitoring of circulation, glucose control, nutritional status, and early signs of impaired tissue repair. Patients receiving inotropic/vasopressor therapy may also require careful monitoring of peripheral perfusion and tissue integrity. This individualized approach is consistent with current international recommendations emphasizing vulnerability-focused rather than uniform HAPI prevention strategies [13,18,26].

Third, the findings highlight the importance of minimizing unnecessary physical restraint use. Although the adjusted association did not reach statistical significance in this study, restraints may still restrict mobility and should be continuously evaluated for clinical necessity. Healthcare teams should consider alternative approaches whenever feasible. Nursing strategies such as improved observation, environmental modification, family participation, delirium prevention, and early mobilization may help minimize reliance on restraints while maintaining patient safety [24,27,28]. Minimizing unnecessary restraint exposure may therefore support both ethical patient care and comprehensive HAPI prevention.

The prevention of HAPI should also be considered a multidisciplinary responsibility. Although nurses play a central role in surveillance and implementation of preventive interventions, successful prevention requires collaboration among physicians, dietitians, rehabilitation specialists, wound care teams, and hospital administrators. Establishing structured HAPI prevention bundles, continuous staff education, clinical auditing, and feedback systems may support adherence to evidence-based practices and may help reduce preventable hospital-acquired HAPI events [13,18,26].

The use of standardized clinical assessment tools and digital health technologies may provide additional opportunities to support HAPI prevention in the future. Electronic health record-based algorithms, artificial intelligence approaches, and continuous monitoring systems have demonstrated potential for earlier identification of patients with characteristics associated with increased vulnerability than traditional assessment methods alone [19,22]. Integrating these technologies with nursing workflows may support timely clinical decision-making and facilitate the targeting of preventive interventions toward patients with greater clinical vulnerability.

## 5. Conclusions

In conclusion, this study found that HAPI development among hospitalized patients with elevated clinical vulnerability was associated with a combination of critical illness characteristics, treatment-related exposures, and underlying medical conditions. Rather than establishing causal pathways, the statistical findings identified several factors associated with HAPI in the final multivariable model, including endocrine and metabolic diseases, cardiovascular diseases, mechanical ventilation, inotropic/vasopressor use, physical restraint, and diarrhea. Among these, endocrine and metabolic diseases, mechanical ventilation, inotropic/vasopressor use, and diarrhea were statistically associated with HAPI, whereas cardiovascular diseases showed a borderline association and physical restraint did not reach statistical significance after adjustment. These findings highlight the importance of vigilant bedside surveillance and suggest that incorporating these clinical parameters into routine nursing assessments may support more individualized clinical assessment of vulnerable inpatients. However, the large adjusted odds ratios and wide confidence intervals observed for several factors, together with the limited number of HAPI cases, indicate substantial statistical uncertainty and potential model instability. Therefore, the findings should be interpreted as exploratory associations rather than definitive causal or independent risk estimates. Further prospective and longitudinal studies with larger samples are warranted to determine whether these associations are consistently observed across different hospitalized populations.

## 6. The Strength and Limitation of the Study

This study has several important strengths. First, it evaluated a broad range of demographic, clinical, and treatment-related variables within a multifactorial analytical framework, allowing the identification of factors independently associated with HAPI while accounting for potential confounding. Second, by focusing on hospitalized patients with elevated clinical vulnerability across multiple specialized units, including general medical wards, the stroke unit, and the medical intensive care unit (MICU), this study provides clinically relevant evidence from acute and intensive care settings. These findings may be particularly informative for healthcare settings in Southeast Asia, where context-specific epidemiological evidence on HAPI remains limited. Third, the use of multivariable logistic regression enabled assessment of the independent associations of multiple clinical factors while accounting for their potential overlap. The final multivariable model demonstrated acceptable calibration based on the Hosmer–Lemeshow test; however, the model performance and estimated associations should be interpreted cautiously given the limited number of HAPI cases and the potential for sparse data and model instability.

Several limitations should be acknowledged. First, the single-center, retrospective observational design limits the generalizability of the findings to other healthcare settings and precludes establishing causal relationships between the identified factors and HAPI development. The findings should therefore be interpreted as associations rather than causal effects.

Second, the relatively modest sample size (*n* = 80; 40 cases and 40 controls) and the limited number of HAPI cases raise concerns regarding the stability of the multivariable logistic regression estimates and the potential for model overfitting. In particular, several exposure categories were sparse, which may have contributed to imprecise estimates. The final model included six factors, and several associations were characterized by relatively large adjusted odds ratios and very wide confidence intervals, particularly for mechanical ventilation, endocrine and metabolic diseases, inotropic/vasopressor use, and diarrhea. These findings indicate substantial uncertainty regarding the magnitude of the observed associations and may reflect sparse-data bias and model instability. Although the final model demonstrated acceptable calibration based on the Hosmer–Lemeshow test, this result should not be interpreted as evidence that the model is free from overfitting or estimation instability. Accordingly, the adjusted estimates should be interpreted cautiously and require confirmation in studies with larger numbers of HAPI events.

Third, because cases and controls were recruited from multiple clinical environments, including general medical wards, the stroke unit, and the MICU, differences in baseline illness severity and intensity of care may have introduced residual confounding. Although the multivariable analysis included selected indicators of severe clinical illness, such as mechanical ventilation and inotropic/vasopressor use, several potentially important factors were not available in the medical records or could not be adequately quantified. These included formal illness severity scores, duration of mechanical ventilation, frequency and consistency of repositioning, nurse-to-patient ratios, and adherence to pressure injury prevention protocols. Unmeasured differences in these factors may have influenced the observed associations.

Fourth, limitations related to the temporal assessment of exposures should be acknowledged. Although a predefined chronological data extraction protocol using electronic medical record timestamps was applied to ensure that recorded exposures preceded the initial identification of HAPI, the retrospective nature of medical record documentation cannot completely exclude documentation delays or minor temporal misclassification. Therefore, the temporal ordering of some exposures may not have been established with the same certainty as in a prospective study. The observed associations, particularly their estimated magnitude, should consequently be interpreted with caution and should not be considered evidence of causality.

Finally, some clinical variables were assessed as broad categories rather than according to their specific severity, duration, or cumulative exposure. For example, mechanical ventilation, inotropic/vasopressor use, diarrhea, and endocrine or metabolic diseases may encompass heterogeneous clinical circumstances that could have different relationships with HAPI development. The limited sample size prevented detailed subgroup analyses of these conditions. Future studies with larger samples should examine the dose, duration, timing, and severity of these exposures to better characterize their relationships with HAPI development.

Future research should prioritize prospective, multicenter studies with larger numbers of HAPI events and standardized longitudinal assessment of patient conditions, illness severity, nursing interventions, and organizational factors. Studies evaluating targeted prevention strategies for mechanically ventilated patients, restraint reduction initiatives, moisture management protocols, and multidisciplinary HAPI prevention bundles are particularly warranted. Furthermore, the integration of artificial intelligence, risk stratification analytics, and real-time monitoring technologies may provide opportunities to improve early identification of clinical vulnerability and support individualized prevention strategies.

## Figures and Tables

**Table 1:** Comparison of Demographic and Clinical Characteristics between the Hospital-acquired pressure injuries and Non-Hospital-acquired pressure injuries Groups (*n* = 80).

Factors	HAPI Group (*n* = 40)	Non-HAPI Group (*n* = 40)	Total (*n* = 80)
	*n* (%)	*n* (%)	*n* (%)
1. Gender			
- Male	25 (62.50)	22 (55.00)	47 (58.80)
- Female	15 (37.50)	18 (45.00)	33 (41.20)
2. Education			
- No Formal Education	1 (2.50)	2 (5.00)	3 (3.80)
- Primary School or Higher	39 (97.50)	38 (95.00)	77 (96.20)
3. Occupation			
- Employed	20 (50.00)	21 (52.50)	41 (51.20)
- Unemployed	20 (50.00)	19 (47.50)	39 (48.80)
4. Marital Status			
- Married/Partnered	33 (82.50)	21 (52.50)	54 (67.50)
- Single/No Partner	7 (17.50)	19 (47.50)	26 (32.50)
5. Income (THB)			
- ≤3078	14 (35.00)	12 (30.00)	26 (32.50)
- >3078	26 (65.00)	28 (70.00)	54 (67.50)
- Mean ± S.D.	5580 ± 4967.55	6930 ± 5479.34	6255 ± 5240.71
- Min, Max	700, 25,000	700, 20,000	700, 25,000
6. Underlying Diseases			
- Present	33 (82.50)	35 (87.50)	68 (85.00)
- Absent	7 (17.50)	5 (12.50)	12 (15.00)
7. Braden Score (At Admission)			
- Mean ± S.D.	13.6 ± 4.18	15.55 ± 3.74	14.01 ± 2.63
- Min, Max	8, 23	8, 23	8, 23
8. Braden Score (At Data Collection Date)			
- Mean ± S.D.	12.3 ± 2.43	15.73 ± 1.88	14.01 ± 2.76
- Min, Max	8, 16	10, 18	8, 23
9. Risk Level of Pressure Injury (At Admission)			
- >17 (Low Risk)	8 (20.00)	13 (32.50)	21 (26.20)
- ≤17 (At Risk)	32 (80.00)	27 (67.50)	59 (73.80)
10. Risk Level (At Data Collection Date)			
- >17 (Low Risk)	1 (2.50)	7 (17.50)	7 (8.80)
- ≤17 (At Risk)	39 (97.50)	33 (82.50)	73 (91.20)
11. Level of Consciousness			
- Conscious	32 (80.00)	39 (97.50)	71 (88.80)
- Unconscious	8 (20.00)	1 (2.50)	9 (11.20)
12. BMI (kg/m^2^)			
- <23	21 (52.50)	21 (52.50)	42 (52.50)
- ≥23	19 (47.50)	19 (47.50)	38 (47.50)
- Mean ± S.D.	23.46 ± 4.89	23.44 ± 6.44	23.45 ± 5.68
- Min, Max	16.87, 36.25	9.91, 40.00	9.91, 40.00
13. Primary Diagnosis			
- Respiratory Diseases	18 (45.00)	19 (47.50)	37 (46.20)
- Musculoskeletal Diseases	2 (5.00)	1 (2.50)	3 (3.80)
- Digestive, Liver, and Biliary Diseases	10 (25.00)	4 (10.00)	14 (17.50)
- Endocrine and Metabolic Diseases	4 (10.00)	1 (2.50)	5 (6.20)
- Cardiovascular Diseases	8 (20.00)	4 (10.00)	12 (15.00)
- Hematological Diseases	1 (2.50)	2 (5.00)	3 (3.80)
- Renal and Urinary Tract Diseases	10 (25.00)	13 (32.50)	23 (28.70)
- Cerebrovascular Diseases	4 (10.00)	3 (7.50)	7 (8.80)
- Neurological Diseases	5 (12.50)	1 (2.50)	6 (7.50)
- Infectious Diseases	24 (60.00)	21 (52.50)	45 (56.20)

**Table 2:** Comparison of Mobility and Activity Factors between the Hospital-acquired pressure injuries and Non-Hospital-acquired pressure injuries Groups (*n* = 80).

Factors	HAPI Group (*n* = 40)	Non-HAPI Group (*n* = 40)	Total (*n* = 80)
	*n* (%)	*n* (%)	*n* (%)
1. Length of Hospital Stay (Days)			
- <8 days	11 (27.50)	16 (40.00)	27 (33.80)
- ≥8 days	29 (73.30)	24 (60.00)	53 (66.20)
- Mean ± S.D.	17.25 ± 12.98	14.40 ± 28.52	15.83 ± 22.06
- Min, Max	3, 48	3, 186	3, 186
2. Intravenous Sedation			
- Not Received	37 (92.50)	40 (100.00)	77 (96.20)
- Received	3 (7.50)	0 (0.00)	3 (3.80)
3. Intravenous Analgesics			
- Not Received	31 (77.50)	40 (100.00)	71 (88.80)
- Received	9 (22.50)	0 (0.00)	9 (11.20)
4. Physical Restraint			
- Not Received	16 (40.00)	33 (82.50)	49 (61.20)
- Received	24 (60.00)	7 (17.50)	31 (38.80)
5. ADL Score			
- Mean ± S.D.	3.05 ± 2.76	6.30 ± 5.46	4.68 ± 4.63
- Min, Max	0, 11	0, 20	0, 20
6. ADL Dependency Level			
- Dependent (<12 points)	40 (100.00)	34 (85.00)	74 (92.50)
- Independent (≥12 points)	0 (0.00)	6 (15.00)	6 (7.50)

**Table 3:** Comparison of Oxygen Perfusion Factors between the Hospital-acquired pressure injuries and Non-Hospital-acquired pressure injuries Groups (*n* = 80).

Factors	HAPI Group (*n* = 40)	Non-HAPI Group (*n* = 40)	Total (*n* = 80)
	*n* (%)	*n* (%)	*n* (%)
1. Mechanical Ventilator Use			
- Used	31 (77.50)	3 (7.50)	34 (42.50)
- Not Used	9 (22.50)	37 (92.50)	46 (57.50)
2. Inotropic/Vasopressor Drugs			
- Received	11 (27.50)	3 (7.50)	14 (17.50)
- Not Received	29 (72.50)	37 (92.50)	66 (82.50)
3. Mean Arterial Pressure (mmHg)			
- ≥65	40 (100.00)	40 (100.00)	80 (100.00)
- <65	0 (0.00)	0 (0.00)	0 (0.00)
- Mean ± S.D.	91.55 ± 15.06	94.08 ± 16.33	92.81 ± 15.66
- Min, Max	68, 134	65, 129	65, 134
Comorbidities			
4. Diabetes Mellitus			
- Present	12 (30.00)	13 (32.50)	25 (31.20)
- Absent	28 (70.00)	27 (67.50)	55 (68.80)
5. Vascular Disease			
- Present	20 (50.00)	20 (50.00)	40 (50.00)
- Absent	20 (50.00)	20 (50.00)	40 (50.00)
6. Capillary Blood Glucose (mg%)			
- ≤180	31 (77.50)	31 (77.50)	62 (77.50)
- >180	9 (22.50)	9 (22.50)	18 (22.50)
- Mean ± S.D.	166.75 ± 69.88	150.69 ± 79.32	158.72 ± 74.72
- Min, Max	55, 419	70, 509	55, 509
7. Smoking History			
- Smoker	25 (62.50)	17 (42.50)	42 (52.50)
- Non-smoker	15 (37.50)	23 (57.50)	38 (47.50)
8. Hemoglobin Level (g/dL)			
- ≤11	23 (57.50)	26 (65.00)	49 (61.20)
- >11	17 (42.50)	14 (35.00)	31 (38.80)
- Mean ± S.D.	10.36 ± 2.43	10.18 ± 2.80	10.27 ± 2.61
- Min, Max	4.60, 14.70	4.90, 16.20	4.60, 16.20

**Table 4:** Comparison of Skin Integrity and Nutritional Factors between the Hospital-acquired pressure injuries and Non-Hospital-acquired pressure injuries Groups (*n* = 80).

Factors	HAPI Group (*n* = 40)	Non-HAPI Group (*n* = 40)	Total (*n* = 80)
	*n* (%)	*n* (%)	*n* (%)
1. Age (Years)			
- >60 years	26 (65.00)	27 (67.50)	53 (66.20)
- ≤60 years	14 (35.00)	13 (32.50)	27 (33.80)
- Mean ± S.D.	66.75 ± 12.31	62.98 ± 17.11	64.86 ± 14.92
- Min, Max	41, 87	19, 85	19, 87
2. Body Temperature (°C)			
- ≥37.5	22 (55.00)	15 (37.50)	37 (46.20)
- <37.5	18 (45.00)	25 (62.50)	43 (53.80)
- Mean ± S.D.	37.67 ± 1.16	37.28 ± 1.06	37.48 ± 1.12
- Min, Max	35.80, 41.50	35.00, 39.40	35.00, 41.50
3. Edema			
- Present	25 (62.50)	13 (32.50)	38 (47.50)
- Absent	15 (37.50)	27 (67.50)	42 (52.50)
4. Urinary Incontinence			
- Present	38 (95.00)	30 (75.00)	68 (85.00)
- Absent	2 (5.00)	10 (25.00)	12 (15.00)
5. Fecal Incontinence			
- Present	30 (75.00)	13 (32.50)	43 (53.80)
- Absent	10 (25.00)	27 (67.50)	37 (46.20)
6. Serum Albumin (g/dL)			
- ≤3.5	39 (97.50)	33 (82.50)	72 (90.00)
- >3.5	1 (2.50)	7 (17.50)	8 (10.00)
- Mean ± S.D.	2.45 ± 0.44	2.92 ± 0.58	2.68 ± 0.56
- Min, Max	1.40, 3.70	1.70, 4.20	1.40, 4.20
7. Oral Intake Status			
- NPO	26 (65.00)	3 (7.50)	29 (36.20)
- Normal Intake	14 (35.00)	37 (92.50)	51 (63.80)

**Table 5:** Bivariate Analysis of Factors Associated with Hospital-Acquired Pressure Injuries (*n* = 80).

Factors	HAPI Group (*n* = 40)	Non-HAPI Group (*n* = 40)	Crude OR	*p*-Value
1. Marital Status				
- Married/Partnered	33 (82.50%)	21 (52.50%)	4.26	0.004 *
- Single/Widowed/Divorced	7 (17.50%)	19 (47.50%)		
2. Braden Scale (Risk Level)				
- At Risk (≤17)	39 (97.50%)	33 (82.50%)	8.27	0.011 *
- Low Risk (>17)	1(2.50%)	7 (17.50%)		
3. Mechanical Ventilator Use				
- Used	31 (77.50%)	3 (7.50%)	42.48	<0.001 **
- Not Used	9 (22.50%)	37 (92.50%)		
4. Physical Restraint				
- Received	24 (60.00%)	7 (17.50%)	7.07	<0.001 **
- Not Received	16 (40.00%)	33 (82.50%)		
5. Fecal Incontinence				
- Present	30 (75.00%)	13 (32.50%)	6.23	<0.001 **
- Absent	10 (25.00%)	27 (67.50%)		
6. Oral Intake Status				
- NPO (Nothing by Mouth)	26 (65.00%)	3 (7.50%)	22.90	<0.001 **
- Normal Intake	14 (35.00%)	37 (92.50%)		
7. Edema				
- Present	25 (62.50%)	13 (32.50%)	3.46	0.013 *
- Absent	15 (37.50%)	27 (67.50%)		
8. Level of Consciousness				
- Unconscious	8 (20.00%)	1 (2.50%)	9.75	0.031 *
- Conscious	32 (80.00%)	39 (97.50%)		
9. Serum Albumin Level				
- Low (≤3.5 g/dL)	39 (97.50%)	33 (82.50%)	8.27	0.038 *
- Normal (>3.5 g/dL)	1 (2.50%)	7 (17.50%)		
10. Inotropic/Vasopressor Use				
- Received	11 (27.50%)	3 (7.50%)	4.68	0.037 *
- Not Received	29 (72.50%)	37 (92.50%)		

* *p* ≤ 0.05, ** *p* ≤ 0.001.

**Table 6:** Multivariable Logistic Regression Analysis of Factors Associated with Hospital-Acquired Pressure Injuries (*n* = 80).

Factors	HAPI Group *n* (%)	Non-HAPI Group *n* (%)	Crude OR	*p*-Value	AOR	*p*-Value	95% CI of AOR
1. Endocrine and Metabolic Diseases							
- Yes	4 (10.00)	1 (2.50)	4.33	0.199	25.92	0.046 *	1.06–633.10
- No	36 (90.00)	39 (97.50)	1		1		
2. Cardiovascular Diseases							
- Yes	8 (20.00)	4 (10.00)	2.25	0.218	18.78	0.050	1.00–354.36
- No	32 (80.00)	36 (90.00)	1		1		
3. Physical Restraint							
- Received	24 (60.00)	7 (17.50)	7.07	0.000	5.40	0.084	0.80–36.66
- Not Received	16 (40.00)	33 (82.50)	1		1		
4. Mechanical Ventilator Use							
- Used	31 (77.50)	3 (7.50)	42.48	0.000	104.79	<0.001 **	11.78–932.05
- Not Used (ref)	9 (22.50)	37 (92.50)	1		1		
5. Inotropic/vasopressor use							
- Yes	11 (27.50%)	3 (7.50%)	4.68	0.037 *	17.26	0.039 *	1.15–258.41
-No	29 (72.50%)	37 (92.50%)	1		1		
6. Diarrhea							
- Yes	9 (22.50)	2 (5.00)	5.51	0.037	24.34	0.015 *	1.88–315.36
- No (ref)	31 (77.50)	38 (95.00)	1		1		

* *p* ≤ 0.05, ** *p* ≤ 0.001, Hosmer and Lemeshow test χ^2^ = 0.754, *p*-value = 0.993, Nagelkerke R Square = 0.721, Overall Percentage = 87.50%. AOR = adjusted odds ratio; CI = confidence interval.

## Data Availability

The dataset contains sensitive clinical information collected from hospitalized patients. Therefore, due to ethical considerations, participant confidentiality, and institutional privacy regulations, the raw data cannot be made publicly available. However, the data are available from the corresponding author upon reasonable request, subject to approval by the relevant ethics committee and institutional policies.
